# Intracortical facilitation within the migraine motor cortex depends on the stimulation intensity. A paired-pulse TMS study

**DOI:** 10.1186/s10194-018-0897-4

**Published:** 2018-08-09

**Authors:** Giuseppe Cosentino, Salvatore Di Marco, Salvatore Ferlisi, Francesca Valentino, Walter M. Capitano, Brigida Fierro, Filippo Brighina

**Affiliations:** Department of Experimental Biomedicine and Clinical Neurosciences (BioNeC), University of Palermo, Azienda Ospedaliera Universitaria Policlinico “Paolo Giaccone”, Via Del Vespro, 143, 90100 Palermo, Italy

**Keywords:** Migraine without aura, Glutamate, Transcranial magnetic stimulation, Cortical excitability, Motor cortex, paired pulse

## Abstract

**Introduction:**

Connectivity within the primary motor cortex can be measured using the paired-pulse transcranial magnetic stimulation (TMS) paradigm. This evaluates the effect of a first conditioning stimulus on the motor evoked potential (MEP) elicited by a second test stimulus when different interstimulus intervals are used. Aim of the present study was to provide, in patients suffering from migraine without aura (MwoA), additional information on intracortical facilitation (ICF), short intracortical inhibition (SICI), and long intracortical inhibition (LICI), using different intensities of the test stimulus (TS).

**Methods:**

We enrolled 24 patients with episodic MwoA and 24 age- and sex-matched healthy volunteers. Both patients and controls were randomly assigned to two different experimental groups: the first group underwent evaluation of ICF, while in the second group we assessed SICI and LICI. All these measures were assessed by using three different suprathreshold intensities of the TS (110%, 130% and 150% of the resting motor threshold, RMT). Interstimulus intervals (ISIs) of 10 ms were used for testing ICF, while SICI and LICI were carried out by using 2 ms and 100 ms ISIs respectively. All migraine patients underwent the experimental protocol while in the interictal pain-free state.

**Results:**

A main finding of the study was that an increased ICF could be seen in migraineurs as compared to the healthy subjects only by using a 110% intensity of the TS. Instead, no significant differences were observed between patients and controls as regards both measures of intracortical inhibition.

**Conclusion:**

We show that hyperresponsivity of the glutamatergic intracortical circuits can be detected in the migraine motor cortex only by applying a low suprathreshold intensity of stimulation. Our results strengthen the notion that, to be reliable, the assessment of cortical excitability in migraine should always include evaluation of the cortical response to different stimulation intensities.

## Introduction

Impairment of mechanisms regulating the brain’s response to various exogenous and endogenous stimuli is supposed to be at the core of migraine pathophysiology. Although the real nature of migraine ‘dysexcitability’ still remains subject of discussion, there is general agreement that brain abnormalities widely affect both cortical and subcortical areas. Several authors have supposed that a functional imbalance between inhibitory and excitatory intracortical circuits, in favour of this latter, could represent the ‘primum movens’ of migraine pathophysiology [1, 2]. Transcranial magnetic stimulation (TMS) represents a valuable tool for assessment of both inhibitory and excitatory processes within the cerebral cortex. TMS studies targeting the visual cortex have provided evidence of cortical hyperexcitability in migraine [3, 4]. More conflicting findings, however, have been obtained at the level of the motor cortex [1]. Some authors have used the paired-pulse TMS paradigm to test connectivity within the primary motor cortex in migraine sufferers [5–10] This approach is based on the application of a conditioning stimulus through the same coil as a second test stimulus [11]. The conditioning magnetic pulse activates inhibitory and facilitatory intracortical interneurons that project to the cortico-spinal tract modulating its response to a subsequent ipsilateral test shock: the response is inhibited when short interstimulus intervals (ISIs) of 1–5 ms (short intracortical inhibition, SICI) or longer ISIs of 50–400 ms (long intracortical inhibition, LICI) are used, whilst a facilitatory effect (intracortical facilitation, ICF) is seen at intermediate ISIs of 6–30 ms [11–13]. Although some researchers have found an increased ICF [9] or a reduced SICI [6–8] favouring the hypothesis of a cortical hyperexcitability in migraine, others have failed to confirm these results [5, 10]. Besides the disease heterogeneity in the patient population, the use of different stimulation parameters might explain such inconsistencies among studies. Indeed, evidence has been provided that in migraine, various methods of cortical stimulation may induce different, even opposite responses (e.g., hypo- or hyperresponsivity) when applied at different stimulation intensities that induce a different degree of cortical activation [1]. Moreover, as regards the paired-pulse TMS paradigm, it has been shown that changes in the intensity of the test stimulus have a considerable effect on measures of cortical inhibition and facilitation in the healthy subjects [14, 15]. On these bases, the main aim of the present study was to provide additional information on intracortical inhibition (SICI and LICI) and facilitation (ICF) using different intensities of the test stimulus in patients suffering from migraine without aura (MwoA).

## Methods

### Subjects

Twenty-four right-handed patients affected by MwoA (7 males/17 females, mean age 35.3 ± 10.7 SD) and 24 right-handed sex- and age-matched healthy subjects (8 males/16 females, mean age 33.2 ± 13.6 SD) without any family history of migraine participated in the study. All patients were recruited from the Headache Outpatient Service of the Neurology Department at the University of Palermo, Italy. Diagnosis of MwoA was made according to the International Classification of Headache Disorders (ICHD 3rd edition, beta version). Additionally, a daily headache diary was used to substantiate the diagnosis and to assess the headache features for a minimum of 3 months before the patients were enrolled in the study.

None of the patients was taking antimigraine prophylactic drugs at least 3 months prior to the study, and to avoid non-specific effects on cortical excitability female subjects (both patients and controls) were not assessed during the menstrual phase. Patients were examined interictally at least 3 days before and after a migraine attack (we checked for the absence of attacks after the recording by means of a telephone call).

Both patients and control subjects were randomly assigned to two groups, who underwent different experimental procedures (see later). Demographic and clinical data of the two groups are presented in Table 1. All subjects enrolled did not suffer from any systemic, neurological or psychiatric disease, and presented with normal physical and neurological findings.

**Table 1:** Demographic and clinical characteristics and resting motor threshold (RMT) values of the enrolled subjects

	Subjects group 1 (*n* = 12)	Subjects group 2 (n = 12)
Healthy subjects		
Mean age (yrs) ± SD	37.5 ± 16.1	28.8 ± 9.5
Sex (M/F)	4 M/8F	4 M/8F
Resting motor threshold (RMT)	46.6 ± 5.6	47.2 ± 3.5
MwoA patients		
Mean age (yrs) ± SD	34.8 ± 11.3	35.8 ± 10.6
Sex (M/F)	3 M/9F	4 M/8F
Mean attack frequency (attacks/month) ± SD	3.3 ± 2.0	4.0 ± 2.6
Mean attack duration (hours) ± SD	37.7 ± 23.2	28.5 ± 25.6
Mean disease duration (yrs) ± SD	12.4 ± 10.3	11.8 ± 10.4
Resting motor threshold (RMT)	54.7 ± 7.4	49.4 ± 4.5

Abbreviations: *SD* standard deviation, *M/F* male/female

Prior to the experiment all subjects gave their informed consent to participate according to the Declaration of Helsinki. The study was approved by the Local Ethics Committee of the University Polyclinic of Palermo.

### Transcranial magnetic stimulation

All subjects were comfortably seated in a reclining armchair and told to be as relaxed as possible. Focal TMS was applied over the left-hand motor cortex by means of a figure-of-eight coil (double-circular-70-mm coil) connected to two Magstim 200 stimulators through a Bistim module (Magstim Co., Dyfed, UK). The stimulating coil was placed over the optimal site for eliciting responses in the contralateral abductor pollicis brevis (APB) muscle. We used a tight-fitting plastic swimming cap to mark the optimum stimulation site in each subject. The surface electromyography (EMG) signals were recorded using 0.9-cm diameter Ag-AgCl electrodes from the target muscle, with a bandpass of 10 to 1000 Hz and a display gain ranging from 50 to 1000 μV/cm. The EMG signals were collected, averaged, and analyzed off-line. The resting motor threshold (RMT) for eliciting responses in the relaxed APB muscle was defined as the minimum intensity of stimulation needed to produce responses of 50 μV in at least 50% of 10 trials. Stimulation was performed following safety guidelines [16].

### Experimental paradigm and measurements

All subjects were randomly divided into two groups, each of which comprising 12 patients and 12 healthy subjects. In the two experimental groups, we assessed different measures of intracortical excitability in the hand motor cortex of the left hemisphere. The first experimental group underwent assessment of intracortical facilitation (ICF). In the second group we evaluated two different measures of intracortical inhibition, i.e. short intracortical inhibition (SICI), and long intracortical inhibition (LICI). All these measures were assessed by means of a paired-pulse paradigm, in which two magnetic stimuli were given through the same stimulating coil. The intensities of stimulation were expressed as a percentage of the RMT. For both ICF and SICI the conditioning stimulus (CS) was set to a sub-threshold intensity of 80% of the RMT, whilst for LICI we used a supra-threshold intensity of 130% of the RMT. The paired-stimulation paradigm was performed according to a previous study with minor modifications [15].

In order to examine whether changes in the test stimulus intensity had different effects on ICF, SICI and LICI, the testing stimulus (TS) was delivered at three different stimulation intensities: 110%, 130% and 150% of the RMT. In the experiment 1, we tested ICF by performing 60 trials, i.e. 10 test stimuli alone and 10 paired stimulations at ISIs of 10 ms, both applied at three different intensities of the test stimulus (110%, 130% and 150% of the RMT). In the experiment 2, we tested SICI and LICI by performing 60 trials for each measure in a single session. As for ICF, 10 test stimuli alone and 10 paired stimulations were applied for each intensity of the test stimulus, using ISIs of 2 ms and 100 ms respectively. In both experiments, trials were performed in a random order with a 10 s inter-stimuli interval, needed to avoid any interference between two successive pulses. The amplitude of motor evoked potentials (MEPs) elicited by single or paired magnetic stimuli was calculated peak-to-peak and then averaged for each stimulation intensity. The effect of the conditioning stimuli on MEP amplitude was determined as the ratio of the average amplitude of the conditioned MEP to the average amplitude of the unconditioned test MEP. In all the experimental conditions, a ratio less than 1 indicated inhibition, while a ratio greater than 1 indicated facilitation.

### Statistical analysis

The mean values of all electrophysiological measures obtained in single subjects were submitted to statistical analyses. The statistical significance of differences among demographic characteristics and RMT values between patients and controls, as well as of differences among clinical characteristics between the two groups of patients was analyzed using Student’s t test. Two-way repeated-measures analyses of variance (ANOVAs) were performed for ICF, SICI and LICI, using the within-subject factor “Intensity of the test stimulus” (3 levels: 110%, 130% and 150% of the RMT) and the between-subject factor “Group” (patients vs. healthy subjects). If ANOVA showed significant differences, Bonferroni post hoc test was used for multiple comparisons of means. The sphericity assumption was checked by using Mauchly’s test, and Huynh-Feldt’s correction was adopted, if necessary, for the degrees of freedom. Pearson’s test was used to check for correlation of the electrophysiological measures with the clinical and demographic characteristics (i.e., age, attack frequency, attack duration, and disease duration). For all analyses the statistical significance was set at *p* values lower than 0.05. All statistics were calculated with Statistica 7.0 software (StatSoft, Tulsa, OK).

## Results

**Fig. 1:**
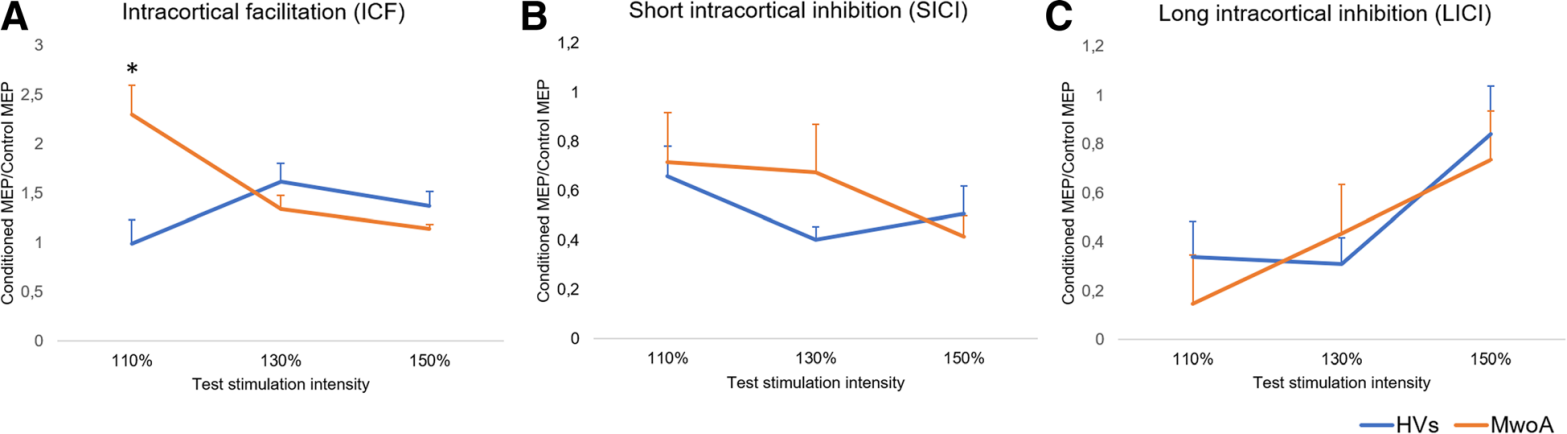
Electrophysiological measures by paired-pulse TMS recorded from migraine without aura (MwoA) patients and healthy volunteers (HVs) after stimulation of the left primary motor cortex. **a** Mean values of intracortical facilitation (ICF) determined as the ratio of the average amplitude of the conditioned MEPs to the average amplitude of unconditioned test MEPs. *Significant variation (*p* = .014) compared to values recorded in the HVs. **b** and (**c**) Mean values of short intracortical inhibition (SICI) and long intracortical inhibition (LICI) determined as the ratio of the average amplitude of conditioned MEPs to the average amplitude of unconditioned test MEPs. Error bars indicate standard errors of means (SEs)

**Fig. 2:**
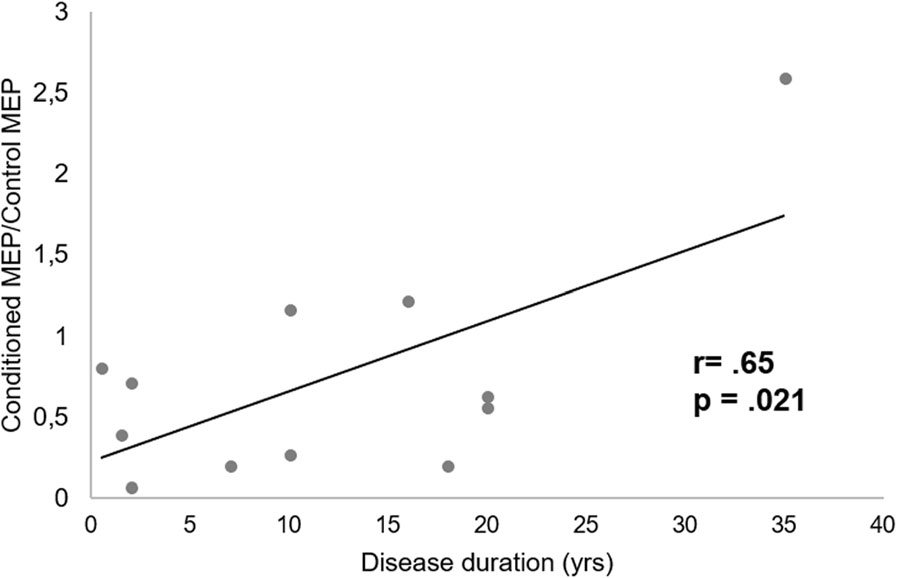
Correlation between individual extent of long intracortical inhibition (LICI) values (ratio of the average amplitude of the conditioned MEPs to the average amplitude of the unconditioned test MEPs) and disease duration (yrs) in migraine without aura patients

The experimental procedures were well tolerated and no adverse effects were reported by any of the participants. No significant differences were found for the demographic and clinical characteristics between the two groups of patients. No significant differences between patients and controls were observed as regards age and RMT (Table 1). F and p values for all ANOVAs are showed in Table 2.  ANOVA used to evaluate ICF (Fig. 1a) showed a significant interaction effect between “Intensity of the test stimulus” and “Group” (F(2, 44) = 15.52; *p* = .00001). Post-hoc analysis showed that ICF assessed at stimulus intensity of 110% of the RMT was more pronounced in the patients group with respect to the healthy volunteers (*p* = .014), whilst no significant differences were observed for the other test stimulation intensities. Only in the patients group we also observed a significant difference between values of ICF assessed at stimulus intensity of 110% as compared to 130% (*p* = .002) and 150% of the RMT (*p* = .001). ANOVA for SICI (Fig. 1b) showed neither significant main effects nor interaction between factors (F(2, 44) = 1.4408, *p* = .24769). Finally, ANOVA for LICI only showed a significant effect of factor “Intensity of the test stimulus” (F(2, 44) = 7.2253, *p* = .00193), in the absence of any significant interaction effect among factors (F(2, 44) = 1.7734, *p* = .18167). The correlation analysis showed no correlation between demographic, clinical and electrophysiological findings in the group of patients that underwent assessment of ICF. Instead, in the subgroup of patients who underwent assessment of intracortical inhibition, we recorded a positive correlation between disease duration and LICI ratios assessed at 150% of the RMT (*r* = .65; *p* = .021) (Fig. 2). This indicates that, with increasing duration of the disease, intracortical inhibition mediated by GABAB receptors decreases when assessed at the higher intensity of stimulation.

**Table 2:** ANOVA results for intracortical facilitation (ICF), short intracortical inhibition (SICI) and long intracortical inhibition (LICI) data in migraine patients and controls

Main effect	cMEP/MEP ratio for ICF		cMEP/MEP ratio for SICI		cMEP/MEP ratio for LICI	
	F values	*p* values	F values	*p* values	F values	*p* values
Group	F_(1,22)_ = 1.81	*p* = 0.19194	F_(1,22)_ = 0.26	*p* = 0.61291	F_(1,22)_ = 0.29	*p* = 0.59267
Stimulation intensity	F_(1.88,41.46)_ = 2.91	*p* = 0.06885	F_(1.98,43.56)_ = 2.32	*p* = 0.11050	F_(2,44)_ = 7.23	*p* = 0.00193
Group x Stimulation intensity	F_(1.88,41.46)_ = 15.52	*p* = 0.00001	F_(1.98,43.56)_ = 1.44	*p* = 0.24779	F_(2,44)_ = 1.77	*p* = 0.18167

*cMEP* conditioned Motor Evoked Potential, *MEP* unconditioned Motor Evoked Potential

## Discussion

To our knowledge, this is the first study to investigate how changes in the stimulation intensity may affect measures of intracortical facilitation and inhibition in the motor cortex of migraine patients. It has been shown that ICF, SICI and LICI may vary in relation to the strength of the test stimulus in the healthy subjects. In particular ICF can be reduced at higher intensities of stimulation, while SICI tends to increase and LICI to decrease with increasing test pulse intensity [15]. It is known that different neuronal populations mediate ICF, SICI and LICI. In particular, electrophysiological studies evaluating the effects of different pharmacological agents on these measures, suggest that glutamatergic circuits are mainly involved in mediating ICF, while SICI and LICI are related to the GABAergic function mediated by the GABAA and GABAB receptors respectively [17]. Findings that measures of intracortical excitability vary in relation to the intensity of stimulation indicate that different neuronal circuits can have different activation and inhibition thresholds or can be spatially recruited in an intensity-dependent fashion. Only a few studies have assessed intracortical excitability in migraineurs by means of the paired-pulse TMS paradigm, providing inconsistent findings likely due to differences in the stimulation parameters used, and because of a lack of evaluation of the intensity-dependent behavior of these measures.

With regard to ICF, Siniatchkin et al. [9] found increased values in migraineurs as compared to the healthy subjects, whilst other authors failed to find such a difference [5–8, 10]. It is noteworthy, however, that only Siniatchkin et al. [9] used suprathreshold intensity for the conditioning stimulus, so making the paradigm more sensitive in detecting changes in ICF. Moreover, it should be also noticed that when using suprathreshold intensities for the conditioning stimulus, that so evokes motor responses in the target muscle, it cannot rule out the possibility that also subcortical structures could be involved in the observed effects.^13^ Main result of the present study is that when using subthreshold intensity for the conditioning stimulus, an increased ICF can be observed in migraine only if a low suprathreshold intensity of the test stimulus is used. This finding of abnormally increased glutamatergic neurotransmission within the migraine motor cortex agrees with other lines of evidence including: (1) results by several other neurophysiological studies [3, 4, 18–22]; (2) detection of higher glutamate levels in plasma, cerebrospinal fluid, platelets and erythrocytes of MwA and MwoA patients as compared to healthy subjects [23–26]; (3) efficacy of antiepileptic drugs acting on the glutamatergic system in the prophylactic treatment of migraine [27, 28]; (4) findings provided by neuroimaging techniques such as magnetic resonance spectroscopy (MRS) studies [29, 30]; and, finally, (5) experimental animal models of familial hemiplegic migraine (FHM) showing that excessive glutamate release mediated by increased presynaptic Ca2+ influx may represent the final common effect of different genetic alterations [31, 32].

Finding that increased ICF cannot be detected in migraine at higher stimulation intensities deserves to be discussed. We recently showed that in migraine with and without aura patients the response to a TMS paradigm consisting of brief trains of 5-Hz repetitive TMS, that normally evoke a progressive MEP potentiation, can induce an inhibitory response at stimulation intensities equal or above to 120% of the RMT [19, 22]. This finding was interpreted in the light of the Bienenstock–Cooper–Munro (BCM) model on cortical homeostatic metaplasticity [33], hypothesizing that in a condition of increased cortical responsivity, like that supposed to be present in migraine, the threshold for the induction of inhibitory cortical responses to an external stimulation could decrease to protect against the risk of neuronal damage. This view was also supported by evidence that inhibitory preconditioning with cathodal transcranial direct current stimulation (tDCS) could restore a normal facilitatory response to the rTMS trains, likely by inducing a decrease in cortical activity, and consequently, according to the BCM model, a weakening of homeostatic mechanisms downregulating cortical excitability [34]. In addition to the above, other kinds of evidence support the role of homeostatic plasticity changes in migraine pathophysiology both as regards recurrence of the attacks and chronification of the disease over time [22, 29, 35]. On these bases, we could suppose that when increasing the test stimulus intensity in the paired-pulse TMS paradigm, inhibitory regulatory mechanisms counteracting cortical hyperresponsivity could be activated when assessing ICF. These could include activation of intracortical inhibitory circuits and/or activity-dependent inhibition of the presynaptic glutamate release, according to the hypothesis that presynaptic mechanisms regulating glutamatergic neurotransmission are involved in cortical metaplasticity [36]. In favor of the latter, we have finding that in the present work we did not observe changes in measures of intracortical inhibition at the different stimulation intensities tested. However, we should consider that protocols for testing intracortical inhibition carry the risk to assess inhibition contaminated by activity of facilitatory intracortical circuits, and not exclusively the activity of the GABAergic circuits [37]. Thus, though measures of intracortical inhibition were not increased with respect to the normal subjects when tested at 130% and 150% of the RMT, the finding that ICF normalized at the higher stimulation intensities could indicate a relative increase of intracortical inhibition leading to restoration of a more physiological balance between motor cortical excitation and inhibition.

An interesting finding of the present study was represented by the positive correlation seen between LICI ratios assessed at intensity of 150% of the RMT and duration of the disease. As higher LICI ratios reflect decreased cortical inhibition, this datum seems to indicate that a progressive weakening of the intracortical inhibitory tone may occur in the course of the disease. This result is in line with previous reports that the cortical silent period (CSP), that represents another measure of GABAB-mediated inhibition within the primary motor cortex, may reduce in migraine patients [38], and this reduction may be associated with an increasing frequency of the migraine attacks, as it may occur in the course of the disease [39].

Some considerations and limitations of the present study should be acknowledged. The first refers to the absence of a group of patients suffering from migraine with aura (MwA). This was mainly due to previous evidence showing no differences between migraine with and without aura patients as regards cortical excitability measures [22, 38, 40–42]. However, it should be also noticed that reduced SICI was found by Brighina et al. [6, 7] in MwA patients, so suggesting that the two pathological conditions cannot be considered entirely comparable. In addition, a decreased SICI was also recently observed by Neverdahl et al. [8] in migraine without aura patients, only apparently in contrast to the present results. Indeed, in this case it is relevant that the authors found reduced SICI only at ISIs of 4 ms, but not at ISIs of 2 ms as those used in the present study. This further supports the great importance of the stimulation parameters used to test cortical excitability in migraine, and the need of standardization in this field of research. Another limitation refers to the fact that all patients were assessed only in the interictal phase, despite it is now clear how cortical excitability significantly changes throughout the various phases of the migraine cycle [8, 22, 43, 44]. Therefore, assessment of cortical excitability in patients during the different stages of the disease or, ideally, in the same patients in different periods of the migraine cycle, is needed to get a wider and clearer understanding of the complex picture of migraine ‘dysexcitability’.

In conclusion, our results strengthen the notion that an intracortical excitation/inhibition imbalance, possibly due to a primary hyperactivity of the glutamatergic intracortical circuits, could represent the pivotal mechanism in migraine pathophysiology. We also provide evidence of a progressive weakening of the intracortical inhibition mediated through GABAB receptors during the course of the disease, that could contribute to evolution of the disease characteristics over time.

## Abbreviations

ANOVA: Analysis of variance
APB: Abductor pollicis brevis
CS: Conditioning stimulus
EMG: Electromyography
ICF: Intracortical facilitation
ISI: Interstimulus interval
LICI: Long intracortical inhibition
MEP: Motor evoked potential
MwA: Migraine with aura
MwoA: Migraine without aura
RMT: Resting motor threshold
SICI: Short intracortical inhibition
TMS: Transcranial magnetic stimulation
TS: Test stimulus
